# Simultaneous Multi-Antibody Staining in Non-Small Cell Lung Cancer Strengthens Diagnostic Accuracy Especially in Small Tissue Samples

**DOI:** 10.1371/journal.pone.0056333

**Published:** 2013-02-13

**Authors:** Gian Kayser, Agnes Csanadi, Claudia Otto, Till Plönes, Nicola Bittermann, Justyna Rawluk, Bernward Passlick, Martin Werner

**Affiliations:** 1 Institute of Pathology, University Hospital Freiburg, Freiburg, Germany; 2 Department of Thoracic Surgery, University Hospital Freiburg, Freiburg, Germany; 3 Department of Hematology and Oncology, University Hospital Freiburg, Freiburg, Germany; Boston University Medical Center, United States of America

## Abstract

Histological subclassification of non-small cell lung cancer (NSCLC) has growing therapeutic impact. In advanced cancer stages tissue specimens are usually bioptically collected. These small samples are of extraordinary value since molecular analyses are gaining importance for targeted therapies. We therefore studied the feasibility, diagnostic accuracy, economic and prognostic effects of a tissue sparing simultaneous multi-antibody assay for subclassification of NSCLC. Of 265 NSCLC patients tissue multi arrays (TMA) were constructed to simulate biopsy samples. TMAs were stained by a simultaneous bi-color multi-antibody assay consisting of TTF1, Vimentin, p63 and neuroendocrine markers (CD56, chromogranin A, synaptophysin). Classification was based mainly on the current proposal of the IASLC with a hierarchical decision tree for subclassification into adenocarcinoma (LAC), squamous cell carcinoma (SCC), large cell neuroendocrine carcinoma (LCNEC) and NSCLC not otherwise specified. Investigation of tumor heterogeneity showed an explicit lower variation for immunohistochemical analyses compared to conventional classification. Furthermore, survival analysis of our combined immunohistochemical classification revealed distinct separation of each entity's survival curve. This was statistically significant for therapeutically important subgroups (p = 0.045). As morphological and molecular cancer testing is emerging, our multi-antibody assay in combination with standardized classification delivers accurate and reliable separation of histomorphological diagnoses. Additionally, it permits clinically relevant subtyping of NSCLC including LCNEC. Our multi-antibody assay may therefore be of special value, especially in diagnosing small biopsies. It futher delivers substantial prognostic information with therapeutic consequences. Integration of immunohistochemical subtyping including investigation of neuroendocrine differentiation into standard histopathological classification of NSCLC must, therefore, be considered.

## Introduction

Lung cancer exhibits the highest overall mortality of malignant disease in humans worldwide [1], [2]. Despite tremendous progress in modern medicine, death rates in lung cancer are still in a steady state reflecting the urgent need of powerful targeted chemotherapeutic agents in the treatment of this widespread disease. Among the separation of small cell lung cancer (SCLC) and non-small cell lung cancer (NSCLC), the latter can be further subclassified according to its morphological appearance into three major groups: adenocarcinomas (LAC), squamous cell carcinomas (SCC) and large cell carcinomas (LCC). Beside different morphology recent studies have revealed biological differences between the histological NSCLC subtypes. These include different expression patterns of m-RNA and immunohistochemically detectable proteins such as thymidylate synthetase (TS). TS, involved in DNA-repair mechanisms, is one of the main targets of pemetrexed and significantly higher expressed in SCC compared to LAC and LCC. Scagliotti et al. could show that patients suffering from LAC and LCC benefit from platin-based chemotherapy in combination with pemetrexed. In contrast, SCC patients benefit from a combination of platin with gemcitabine [3]. Based mainly on this study, recommendations for exact subtyping of NSCLC were introduced into national lung cancer guidelines [4]–[6]. The dilemma for pathologists in this setting is, on the one hand, the current WHO classification introduced in 2004. According to the WHO, subclassification of NSCLC has to be made mainly by hematoxilin-eosin (H&E) stains. Due to often extensive tumor heterogeneity, resection specimens are demanded for a definitive diagnosis, too [2]. On the other hand, these treatment guidelines prevail for patients in advanced UICC-stages IIIB and IV [3]. Of these patients, typically, only small tumor samples are gathered, usually, by biopsy, mediastinoscopy or even fine needle aspiration. Thus, the pathologist needs to base his/her diagnostic decision on additional immunohistochemical special stains. Among others, especially thyroid transcription factor 1 (TTF1) and squamous stem cell marker p63 are recommended in first line [7]–[9]. Since tumor material from small biopsies is very scarce, tissue sparing techniques have to be developed to cope with lesser diagnostic material for a larger number of diagnostic investigations. Additionally, as the quantity of needed molecular investigations for targeted therapy is growing rapidly, economical considerations must also be taken into account when introducing new techniques. Based mainly upon published marker sets, Sterlacci [10] and Yamagita [11] lately published double staining protocols and a commercially available antibody cocktail, respectively. Nevertheless, beside LAC and SCC, large cell neuroendocrine carcinoma (LCNEC) represents another subentity of NSCLC with therapeutic consequences. In recent studies, it has become more and more obvious that LCNEC are biologically closely related to SCLC [12], [13]. It is therefore proposed to administer LCNEC patients chemotherapeutic combinations in analogy to SCLC [14]. In the strategy of NSCLC diagnosis and subtyping, LCNEC should by these means be included. Upon this background we investigated benefit of a newly developed simultaneous multi-antibody staining assay in a large and well characterized cohort of NSCLC patients.

## Materials and Methods

### Ethics statement

The study has been approved by the University Hospital Freiburg (EK 10/12). In concordance with this decision of this ethics committee all patient relating data were used only in a pseudonymous manner. The results obtained by this study did not influence patient's treatment. The paraffin material had been archived at least 3 years after initial diagnosis. Due to these prerequisites, by decision of the ethics committee, no written consent of each patient had to be obtained.

### Patients and TMA construction

265 patients, operated on between January 1^st^ 1990 and December 31^st^ 2007 at the Department of Thoracic Surgery, University Hospital Freiburg, were chosen from the database of the Institute of Pathology, University Hospital Freiburg. Beside primary NSCLC, inclusion criteria were access to sufficient tumor and non-neoplastic lung tissue. Selection of patients was carried out blinded to avoid biases resulting from tumor stage and/or histological subtype. Operation specimens were fixed in 4% buffered formalin for 24 to 48 hours. Grossing procedure and paraffin embedding followed routine protocols. All tumor bearing paraffin blocks were retrieved from the archive. Upon new H&E sections all tumors were reclassified independently by three experienced pathologist (GK, AC, MW) according to the current WHO classification [2]. No immunohistochemical staining results had been taken into consideration for this reclassification. 5 discordant and difficult cases were, again, reviewed and discussed in common. Upon the presence of WHO criteria, consensus was defined for further analyses. TNM-stages were uniformly reassessed following the current UICC classification (7^th^ edition) [15]. To eliminate biases, histological reclassification and UICC restaging of all cases were performed prior to immunohistochemical procedures and their evaluation. From all patients, clinico-pathological data including smoking habits and overall survival were available (table 1).

**Table 1:** Summary of clinico-pathological data of included NSCLC patients.

Age	Median 65 years	
Overall survival	Median 32.5 months	
	Number	Percent
**Sex**		
Male	190	71.7%
Female	75	28.3%
**Smoker**		
No	72	27.2%
Yes	193	72.8%
**pT-stage**		
pT1a	30	11.3%
pT1b	35	13.2%
pT2a	92	34.7%
pT2b	30	11.3%
pT3	60	22.6%
pT4	18	6.8%
**pN-stage**		
pN0	146	55.1%
pN1	40	15.1%
pN2	73	27.5%
pN3	2	0.8%
Not assessable	4	1.5%
**UICC-stage**		
IA	40	15.1%
IB	51	19.2%
IIA	38	14.4%
IIB	30	11.3%
IIIA	84	31.7%
IIIB	8	3.0%
IV	9	3.4%
Not assessable	5	1.9%

Tissue multi-arrays (TMA) were constructed by taking three cores of each tumor at random from three different distant areas. Core diameter was 2 mm. In H&E stained sections of the TMAs all cores were evaluated for the presence of distinct glandular or squamous growth patterns which classified these as LAC or SCC, respectively. TMA cores lacking specific growth patterns were diagnosed as NSCLC not otherwise specified (NOS).

### Immunohistochemistry (IHC)

Two micrometer thick sections of the TMAs were mounted on coated glass slides (Superfrost plus), dewaxed and rehydrated in a descending alcohol row. For antigen retrieval, slides were incubated in citrate buffer (pH 6.1) for 2 minutes using a pressure cooker. Incubation time for the first primary antibodies, vimentin (clone VIM3B4) and TTF1 (clone 8G7G3/1), was 30 minutes at room temperature. Incubation with the first secondary biotinylated antibody followed for 15 minutes. Activation and visualization were performed by alkaline phosphatise coupled streptavidine (AP/Streptavidine; DAKO Real Detection system AP/RED, code K5005). The second part of the multi-antibody stain consisted of a primary antibody directed against p63 (clone 4A4) and a neuroendocrine antibody cocktail targeting Chromogranin A (clone DAK-A3), Synaptophysin (clone SY38) and CD56 (clone 1B6). Incubation time for these antibodies was 30 minutes. A horseradish peroxidase conjugated dextran polymer with coupled antibody molecules was applied and staining was performed by incubation with 3′3′-diamino-benzidine (DAB; DAKO envision FLEX+, code 8012). Finally, slides were counterstained with hematoxilin. The staining procedure was performed on a DAKO autostainer for all slides (detailed step-by-step protocol Document S1).

### Evaluation of the multi-antibody assay

**Figure 1:**
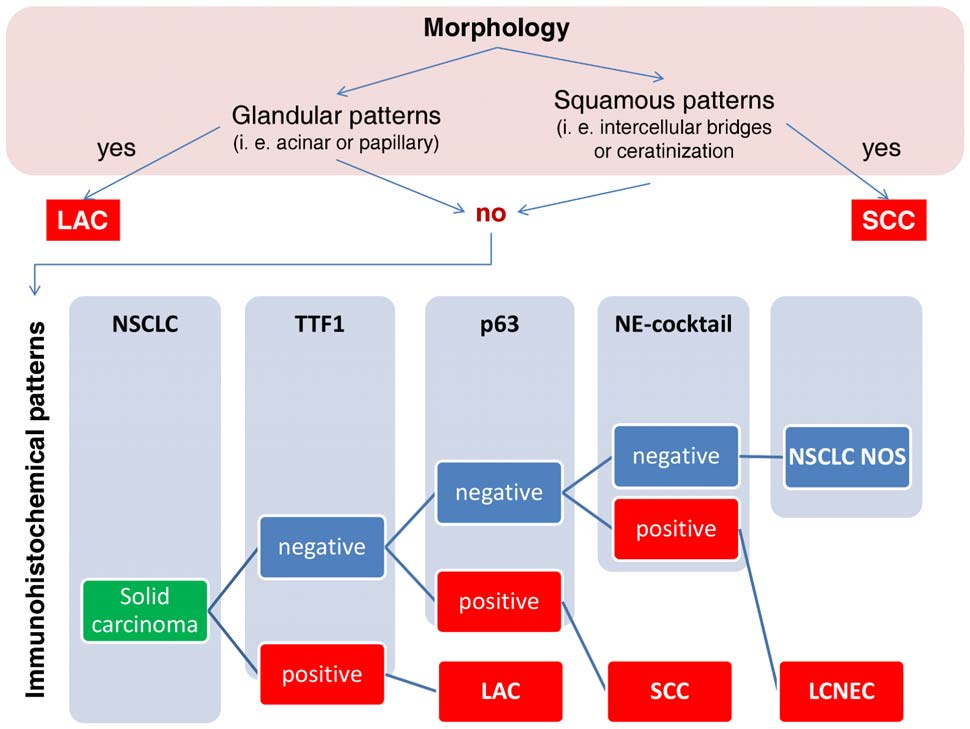
Hierarchical classification of NSCLC using evaluation of growth patterns followed by expression of IHC markers.

In synopsis with the histological growth pattern a combined histological-IHC diagnosis for each core was made in concordance with the proposed IASLC guidelines [9]: Hereby, clear glandular growth patterns defined LAC independent from expression of IHC markers. The same was defined for SCC, if ceratinization and/or intercellular bridges were present. In tumors without definite morphological H&E-diagnosis, evaluation of IHC-marker expression followed this hierarchical algorithm: 1) TTF1 expression: positive LAC, negative: 2) p63 expression: positive SCC, negative: 3) expression of neuroendocrine markers: LCNEC, negative: NSCLC NOS (figure 1). Vimentin served as a surrogate marker to evaluate stromal architecture as well as fixation state of the tissue since its robustness in staining quality. Each marker was evaluated independently, while only distinct specific nuclear (TTF1 and p63) or cytoplasmic (Vimentin, Chromogranin A and Synaptophysin) and membranous (CD56) with at least moderate to strong intensity in the exceeding majority of tumor cells were considered positive for diagnostic purposes.

For final assessment of the tumor, a consensus diagnosis combining all three TMA cores was made. In concordance with the current WHO-classification [15], if the TMA cores were not unanimously diagnosed cancer was classified as LAC for the combination of LAC and NSCLC NOS, and as SCC for the combination of SCC and NSCLC NOS. The combination of LAC or SCC with LCNEC was grouped into LCNEC. If both, LAC and SCC, were diagnosed in different TMA cores of a patient these were classified as mixed LAC/SCC. As interpretation of this IHC expression profile is not clearly defined in the IASLC proposal and only 5 patients grouped into it, these cases were excluded from survival analyses.

### Statistics

Correlation between the different antibodies was evaluated by calculation of the Kendall-tau b correlation coefficient. Diagnostic reliability of the different antibodies for IHC classification algorithm of NSCLC was judged by receiver-operating characteristic (ROC) curves and area under curve (AUC) calculations. Inter-core differences, as well as diagnostic agreement between H&E-classification of resection specimens and definite combined morphological-IHC classification were analyzed by calculation of Cohen's kappa and Cramer's V, respectively. Survival analysis was conducted according to Kaplan-Meier. Log-rank tests served to evaluate statistical significance. Follow-up time was assessed up to 120 months. As published and internationally accepted [16]–[19] 15 patients who died within 30 days of surgery were excluded from survival analyses to exclude bias resulting from perioperative mortality in this long term follow-up study. All statistical analyses were performed using the SPSS 17 software suite. Statistical significance level alpha was set to 5% for all tests.

## Results

### No inter-antibody interactions are observed

**Figure 2:**
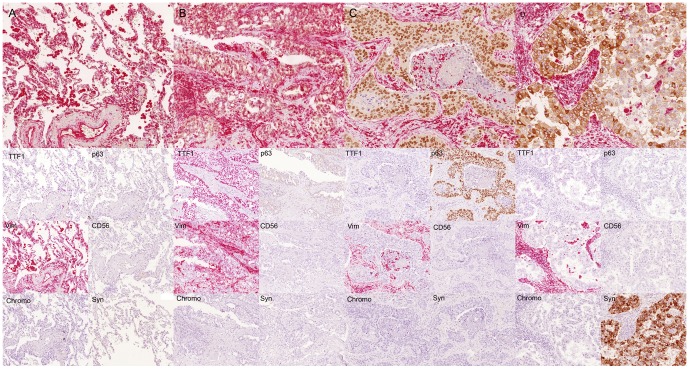
Immunohistological images obtained from multi-antibody assay compared to single antibody stains. Normal lung tissue (A), LAC (B), SCC (C) and LCNEC (D) stained with the combined multi-antibody assay (top) and each single included antibody. Red nuclei – TTF1; red cytoplasm – vimentin; brown nuclei – p63; brown cytoplasm – neuroendocrine markers. Below the bi-color IHC assay corresponding single antibody assays are displayed in the same tumor area. Comparison of each single antibody staining and the bi-color multi antibody assay shows analog specific positivity in the same cells and does not reveal any unspecific background staining or chromogenic cross reactivity.

In order to asses possible interactions between the different antibodies used, a test set of 10 NSCLC and non-neoplastic lung tissue probes was stained using the multi-antibody assay. Serial sections of this test set were stained using only one of the antibodies included in the multi-antibody staining protocol. Reviewing these sections, in all samples no differences in staining patterns could be detected between each single-antibody and the multi-antibody protocol (figure 2). To evaluate possible cross-reactions of the second detection system (DAB-based) with the first applied primary antibody (Vimentin and TTF1), we stained the test set omitting the second primary antibody cocktail (p63, CD56, Chromogranin A and Synaptophysin). These sections only revealed red chromogen staining for Vimentin and TTF1, no cross reaction with the brown DAB based detection system was observed (figure S1.). Furthermore, in analyses including the whole cohort we could not detect any positive statistical correlation between those antibodies used for each chromogenic detection method, i. e. TTF1 and vimentin for AP/Streptavidin and p63 and the neuroendocrine cocktail for DAB. On the contrary, p63 and expression of neuroendocrine markers showed a significant negative correlation (p<0.001; correlation coefficient −0.300; Kendall-tau-b). Significant negative correlation could also be detected for TTF1 and p63 (p<0.001; correlation coefficient −0.262; Kendal-tau-b). No other positive or negative inter-antibody correlations were detected. These findings further support that staining patterns and evaluation of each antibody are not compromised by potential background staining or chromogen cross reactions. Nevertheless, nuclear and/or cytoplasmic double positivity reflecting specific staining for both antigens, i. e. TTF1 combined with p63 and vimentin combined with neuroendocrine markers, respectively, are still clearly distinguishable (figure S2).

### TTF1 and p63 are useful markers for assessment of lineage differentiation

The current WHO classification proposes H&E classification of resection specimens as the gold standard of lung cancer [2]. Regarding this, we could observe a sensitivity and specificity of TTF1 for recognition of LAC in a range in between the three TMA cores of 80.4% to 84.6% and 70.6% to 76.9%, respectively. Evaluating p63 as a marker for squamous differentiation, sensitivity was calculated from 62.1% to 66.7%, whereas specificity ranged between 84.0% and 90.8%. These figures are comparable with the current literature in which these two markers are recommended to be used on small biopsies for investigation of lineage differentiation [8], [9]. This is also reflected in clear cut odd's ratios (figure S3) and the ROC-AUC analyses (figure S4; table S1). Both, TTF1 and p63 revealed highly significant results for classification of LAC and SCC, respectively (p<0.001; table S1). As expected, AUC values were higher in IHC classification algorithm as compared to H&E classification of resection specimens (table S1). In this context we also observed a significant negative correlation between the expression of TTF1 and p63 (overall: p<0.001; correlation coefficient −0.262; Kendall-tau-b).

### Intratumoral heterogeneity is less prominent in IHC markers compared to morphologic growth patterns

As heterogeneity of tumor morphology is known to be high in NSCLC we included three TMA cores of each tumor. These cores were taken at random concerning tumor area to exclude morphological bias. As published and internationally accepted, analysis of each TMA core can be assumed comparable to analysis of tumor biopsies in routine diagnostic pathology [20], [21]. With regard to diagnostic pathways in histopathology, we first classified each TMA core in a routine H&E stain. For evaluation of intra-tumor/inter-core heterogeneity Cohen's kappa was calculated for H&E classification as well as for the combined morphological-IHC classification algorithm. Overall, Kappa-values observed can be interpreted as in agreement with good correlation for all classification algorithms. But results obtained for the combined IHC classification were always at least as good as for H&E inter-core variation (table 2). Currently, IHC algorithms are solely recommended for biopsies which lack specific growth patterns and are therefore classified as NSCLC NOS. Regarding this recommendation, kappa values were separately calculated for those cases in which at least one TMA core was classified as NSCLC NOS upon H&E morphology. Within these cases, kappa-values for H&E classification only showed poor correlation whereas IHC classification reached values of good inter-core correlation (table 2). Analysis of immunohistochemical staining patterns in combination with histological growth patterns for classification of NSCLC, therefore, shows less intratumoral variability than classification of NSCLC based on H&E morphology, alone.

**Table 2:** Inter-core agreement for H&E diagnosis and additional IHC classification algorithm.

All cases			
**Cohen's kappa**	HE – A	HE – B	HE – C
HE – A	*1.000*	0.521	0.548
HE – B		*1.000*	0.643
**Cramer's V**			
HE resection specimen	0.489	0.529	0.544
IHC - classification	0.515	0.515	0.548
**Cohen's kappa**	IHC – A	IHC – B	IHC – C
IHC – A	*1.000*	0.628	0.690
IHC – B		*1.000*	0.752
**Cramer's V**			
HE resection specimen	0.556	0.531	0.547
IHC - classification	0.773	0.748	0.782

Classified as NSCLC in at least one H&E-TMA core			
**Cohen's kappa**	HE – A	HE – B	HE – C
HE – A	*1.000*	0.079	0.009
HE – B		*1.000*	0.321
**Cramer's V**			
HE resection specimen	0.304	0.413	0.352
IHC – classification	0.305	0.335	0.335
**Cohen's kappa**	IHC – A	IHC – B	IHC – C
IHC – A	*1.000*	0.508	0.583
IHC – B		*1.000*	0.677
**Cramer's V**			
HE resection specimen	0.466	0.456	0.477
IHC - classification	0.735	0.725	0.740

Cohen's kappa quantifies the variability of definite H&E diagnosis of the resection specimens and IHC classification algorithm between the different TMA cores. Cramer's V is used to quantify the variability between the TMA cores and the corresponding resection specimens. Both tests reveal a better concordance in IHC-classification compared to H&E classification.

### Additional IHC analyses raise diagnostic accuracy compared to conventional H&E classification

Upon H&E morphology of the TMA cores between 130 and 142 cores of each of the three TMA core sets could not be classified upon distinct growth patterns. These qualified as NSCLC NOS. Combining the three cores to a consensus diagnosis 48 (18.1%) were classified as NSCLC NOS, 14 of which expressed neuroendocrine markers and were therefore diagnosed as LCNEC. In contrast to H&E classification, using the combined IHC algorithm only 13 tumors (4.9%) expressed neither TTF1 nor p63 nor neuroendocrine markers and were therefore classified as NSCLC NOS (table 3). To define diagnostic concordance of each TMA core with histological reclassification of the resection specimen Cramer's V was calculated for each core classified by H&E and the multi-antibody assay. Although we could verify a high statistical significance for the correlation of conventional diagnosis of resection specimen with H&E diagnosis, as well as IHC classification, respectively, overall conformity was always higher by adding the information of our multi-antibody assay (table 3). This is not only reflected in a higher percentage of correctly classified cases but also in a higher value for Cramer's V (table 3).

**Table 3:** Cross-tabulation for concordance or H&E-classification and IHC-classification with classification of resection specimens.

	H&E resection specimen				
TMA-IHC	LAC	SCC	LCC	LCNEC	Total
**LAC**	80	5	4	1	**90**
**SCC**	7	91	26	3	**127**
**LCNEC**	7	4	0	19	**30**
**Mixed LAC/SCC**	3	1	1	0	**5**
**NSCLC**	3	1	8	1	**13**
**Total**	**100**	**102**	**39**	**24**	**265**
**Cramer's V 0.629, p<0.001**					

	H&E resection specimen				
TMA-H&E	LAC	SCC	LCC	LCNEC	Total
**LAC**	90	15	10	4	**119**
**SCC**	4	68	12	4	**88**
**LCNEC**	0	2	4	8	**14**
**Mixed LAC/SCC**	3	3	2	2	**10**
**NSCLC**	3	14	11	6	**34**
**Total**	**100**	**102**	**39**	**24**	**265**
**Cramer's V 0.506; p<0.001**					

Combining all TMA cores to a single diagnosis IHC-classification shows higher concordance with the diagnosis made upon the resection specimen as consensus H&E diagnosis of the TMA cores.

### Inclusion of neuroendocrine markers into routine IHC panel delivers clear definition of LCNEC

Expression of neuroendocrine markers was considered if at least one TMA core expressed specific staining. Comparison of presence and absence of expression of neurendocrine marker did not have an impact on patient's survival analyzing the whole cohort (p = 0.208). This was also true in the survival analyses for LAC (p = 0.690) and SCC (p = 0.207). On the other hand, investigating those NSCLC neither having distinct adenoid or squamous morphological features nor expressing TTF1 or p63, thus being classified as NSCLC NOS, expression of neuroendocrine markers indeed revealed a distinct separation of the obtained survival curves with a statistical trend (p = 0.095; figure S5). On morphological evaluation, all of these LCNEC cases additionally showed neuroendocrine growth patterns. By use of combined IHC classification LCNEC can therefore be diagnosed in a sophisticated and standardized way.

### Combined morphological and multi-antibody classification in enhances clinical significance of NSCLC subtyping

**Figure 3:**
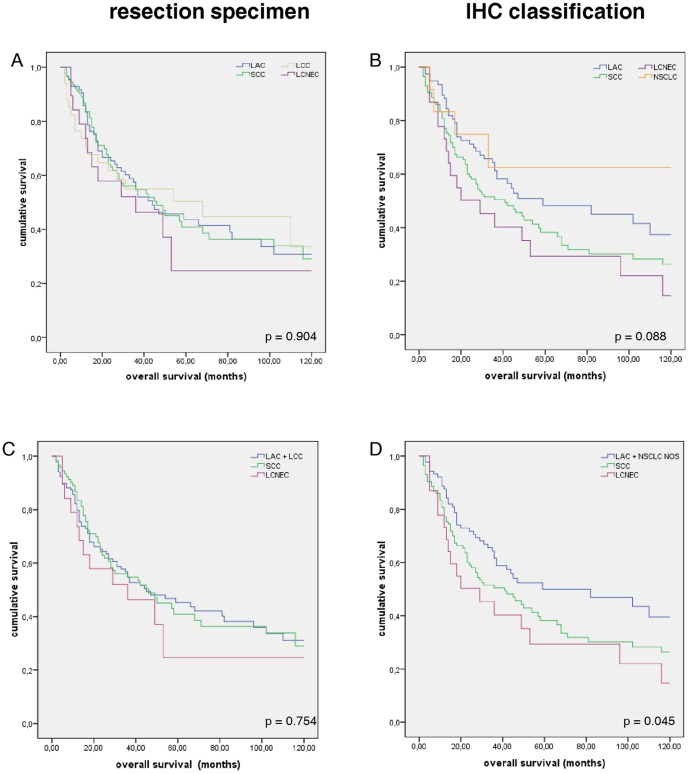
Kaplan-Meier survival curves for histological NSCLC subtypes. NSCLC subtypes were diagnosed by H&E on resection specimens (A) and by combined IHC classification algorithm (B). Additional IHC classification separates distinct histological entities suggesting different biological behavior. Grouping the different entities according to therapeutic consequences (C and D), no differences in survival are evident by conventional H&E classification of resection specimens (C), whereas combined IHC classification significantly separates the different survival curves (p = 0.045; D).

Different biological behavior of malignant diseases is usually reflected in different outcomes in long term follow up. To assess if there is a better stratification by using a combined histological and IHC classification, we conducted survival analyses for both classifications, i. e. conventional H&E morphology of resection specimens and combined morphology-IHC classification algorithm. Conventional, H&E based classification of our cohort resulted in near to parallel Kaplan-Meier survival curves (p = 0.904; figure 3). On the other hand, morphology-IHC classification gives a clearer separation of the survival curves of each entity with a statistical trend (p = 0.088, figure 3). Combining LAC and NSCLC NOS according to therapeutic consequence into “non-squamous carcinoma” and comparing these with SCC and LCNEC overall survival was significantly different between these patient groups (p = 0.045, figure 3).

### Bi-color multi-antibody assay saves tissue and economic resources

The protocol used here to immunohistochemically classify NSCLC consists of a simultaneous stain with 6 different antibodies. Thus, the amount of tissue needed for characterizing NSCLC biopsies comprises only 16.7% in comparison to that needed for 6 single antibody assays. Consequently, only 1/6^th^ of glass sildes and coverslips are needed. Since the protocol uses antibody mixtures of TTF1 and Vimentin, as well as p63 in combination with the neuroendocrine cocktail, 1/3^rd^ of staining and blocking reagents are needed. The same holds true for chromogenic detection vials. At our institution, expenses for reagents are calculated to 3.30 € plus additional 0.20 € for the glass slide per IHC stain. In figures per case, this results in a total amount of 6.80 € for our multi-antibody assay compared to 21.80 € for 6 single antibody protocols. Thus, usage of our multi-antibody assay saves up to 68.8% of expenses for laboratory reagents.

Due to the doublestaining procedure however, overall time needed to complete the protocol takes approximately 4 hours as compared to 1.5 to 2 hours for single antibody stains. But, as the protocol can easily be established on automated immunohistochemical staining machines, no negative effect on the workload of laboratory technicians occurs. On the contrary, staining automation is prolonged and technicians can accomplish more complex and time consuming tasks during this procedure. Additionally, time for slide sorting is completely omitted.

For the diagnosing pathologist only one slide needs to be evaluated. Hereby, all IHC staining patterns can be assessed in parallel and even more importantly in the same cells. Microscopic slides need not to be changed and diagnostic areas not to be relocated. As diagnostic evaluation times in diagnostic pathology are very variable, the amount of time saved by using our multi-antibody assay can only be estimated. According to our experiences the multi-antibody assay saves approximately two thirds of the time needed for evaluation of the corresponding 6 single antibody stains.

## Discussion

Worldwide, lung cancer is the major cause of death among all malignant diseases [1], [22]. Although there have been advances in modern cancer therapy, mortality rates of NSCLC patients have not changed significantly over the last decades [1], [2], [22]. With developing research in personalized cancer management, several biomarkers have been attributed a predictive value for specific chemotherapeutic agents [23]–[27]. Scagliotti et al. described a benefit of gemcitabine in a platin-based chemotherapeutic regime for patients suffering from advanced NSCLC if histology showed a predominant squamous differentiation. In addition, patients whose histology showed a predominant LAC differentiation had a greater benefit if given pemetrexed [3]. Consequently, recommendations for chemotherapeutic regimes are now based on histological differentiation of NSCLC and have been implemented in several national NSCLC treatment guidelines [4]–[6]. In the current WHO classification, histological typing of lung cancer is 1) largely based on standard H&E staining and 2) requires histopathological examination of resection specimens, especially having in mind that almost 50% of lung cancers exhibit more than one major histological subtype [2]. On the other hand, the work of Scagliotti is based on a cohort of NSCLC patients diagnosed in advanced UICC stages IIIB and IV. Here, histological typing has to be made on small tissue samples, as mostly resection specimens are not available in these patients. Therefore, IHC markers and panels have been proposed by several authors for subtyping of NSCLC [8], [28], [29]. Among these, TTF1 for detection of glandular and p63 for squamous differentiation seem to be the most reliable markers for subclassification of NSCLC [8], [9]. For these reasons, we chose TTF1 and p63 as markers for lineage differentiation in NSCLC, and established a diagnostic path combining conventional morphology and IHC expression patterns in analogy to the proposal of the IASLC [9]. Lately, the truncated p63 protein DeltaNp63 (p40) has been described to have a higher specificity for squamous differentiation compared to the full length protein [XPATH ERROR: unknown variable "start2".], [31]. But both antigens, full length p63 and DeltaNp63, have the same sensitivity for squamous cell carcinoma [30], [31]. Since expression of p63 is diagnostically evaluated after TTF1 (figure 2), it can be assumed that both antibodies would give the same classification results in our multi-antibody assay. To include LCNEC, we expanded our antibody panel not only with vimentin, which can serve as a marker for sarcomatoid carcinoma [8], but also with neurendocrine markers (CD56, Synaptophysin, Chromogranin A). Positivity of one or more of these markers is required for the diagnosis of neuroendocrine differentiation [2]. In order to spare the finite tissue of bioptic material, we successfully implemented a bi-color multi-antibody staining assay which allows not only simultaneous staining of 6 antibodies on the same tissue section, but also veritable simultaneous evaluation. To demonstrate that there are no interferences between the antibodies, several validation experiments have been made: A test set was stained with our multi-antibody assay and serial sections have been stained with each antibody, individually. Here, no differences in staining patterns or chromogen background reactions could be observed (figure 2, figure S1). To investigate possible cross-staining by the second DAB-based detection system the assay was performed on the test set while omitting the second primary antibodies (p63, Chromogranin A, Synaptophysin, CD56). If free and/or accessible binding sites of the first antibodies were existing, these would be visible in brown color. Reviewing the stained slides, these experiments prove that all binding sites of the first primary antibodies (Vimentin, TTF1) are blocked by the AEC-based detection system as no brown DAB staining was visible (figure S1). Neither could we elucidate any positive statistical correlation between the antibodies being visualized by the same chromogenic detection method. If there were background staining, either nuclear or cytoplasmic of corresponding markers leading to false interpretation, this would have been reflected in positive statistical correlations. On the contrary, we detected statistically significant negative correlation of p63 with neuroendocrine markers, as well as with TTF1. Recently, Sterlacci et al established double stains consisting of TTF1/CK7, p63/CK5/6 and TTF1/CK5/6 for classification of NSCLC and revealed results similar to ours [10]. Yanagita et al. have published a commercially available multi-antibody assay containing 4 antibodies, TTF1, napsin A, p63 and CK5/6 [11]. These previously established antibody cocktails prove to be useful, but only serve to distinguish LAC from SCC. The possibility to investigate for LCNEC is not taken into account. However, especially this entity of LCNEC not only inhabits an infavourable prognosis, but chemotherapeutic regimes similar to those used in SCLC have to be considered [14]. In the current literature, LCNEC are only to be diagnosed if NSCLC show neuroendocrine growth patterns in combination with expression of at least one of the neuroendocrine markers CD56, chromogranin A or synaptophysin [2]. These recommendations are based upon several studies where expression of neuroendocrine markers did not show significant differences in LAC or SCC but not discriminating the expression of neuroendocrine markers in NSCLC NOS or LCC [12], [13], [32], [33]. Rounding up this diagnostic gap, we are able to demonstrate that classification of LCNEC by using a combined morphological-IHC classification algorithm, as proposed here, may be helpful to consistently identify this entity. This is further supported by the fact that all LCNEC exhibited neuroendocrine growth patterns, as well. In consistency with La Rosa et al who describe 30% of LCNEC to be positive for TTF1 [34] we could observe some TTF1 positivity in 9 out of the 30 LCNEC. Nevertheless, this positivity was usually weak and/or not detected in the exceeding majority of tumor cells. Therefore, this was not regarded as diagnostic for LAC using our modified morphological and immunohistochemical classification algorithm. For these reasons, we opine the combined evaluation for LCNEC in small tissue samples important, not only for accurate diagnosis, but also for therapy stratification. We, thus, demonstrate for the first time the feasibility of a bi-color 6-antibody immunohistochemical assay for accurate and tissue sparing classification of NSCLC. Additionally, our multi-antibody assay is the first to include the possibility for the diagnosis of LCNEC in a tissue and time sparing manner.

Furthermore, as tumor content particularly of endoscopically gathered material can vary greatly [35], and modern pathological diagnostics also include further investigations such as molecular mutation analyses [4]–[6], [24], [36], pathologists are forced to struggle with lesser tissue for increasing diagnostic purposes. Since, compared to single antibody stains, only 1/6^th^ of the tissue is needed for valid subtyping of NSCLC using our proposed multi-antibody assay, it safely addresses this issue. Simultaneous multi-antibody assays not only safe sparse tissue, but also economic resources: In our assay only 17% of glass slides, and 33% of staining reagents are needed. This results in reduction of reagent expenses of 68.8% calculated at our institution. From the technician side, although the staining procedure takes approximately 1 to 1 ½ hours longer, no negative effect on their workload was observed. Our assay can easily be implemented on commercially available automated staining machines. Additionally, taking into account that the multi-antibody assay completes 6 single stains at the same time and on the same slide, no time for coordination of separate staining protocols or for slide sorting is needed. Diagnostic time for evaluation of simultaneous multi-antibody stains also decreases, as pathologists can not only evaluate 6 antibodies at the same time without changing histological slides but also assess all 6 antibodies within the same cells. This avoids misinterpretation of artifacts and searching for the same small tumor area included in the specimen. Since time needed for diagnostic evaluation varies not only from specimen to specimen but also from pathologist to pathologist, savings in this regard are difficult to quantify. According to the experiences, we gathered from the current investigation, it can be estimated that evaluation of our multi-antibody assay takes only around one third of the time needed for diagnostic appraisal of 6 individual antibody stains. Implementation of multi-antibody assays, thus, not only has a tremendous positive impact on saving tissue, but also on conserving considerable economic and diagnostic resources.

To simulate the clinical approach of bioptically collected tissue samples, we used TMAs with a core diameter of 2 mm randomly taken from tumor paraffin blocks. This approach can be considered similar to endobronchial sampling as 1) endobronchial biopsies are not guided by histomorphology and, thus, are also taken from a histomorphologically random tumor area and 2) tissue specimens gathered by current endobronchial techniques deliver comparable sample sizes [37]. It can therefore be assumed that simulation of bronchial biopsies by the use of TMAs with an adequate core diameter delivers equal results and is comparable to the clinical-histopathological setting. In several publications, simulating bioptic specimens by TMA cores has also been internationally accepted [20], [21]. Nevertheless, this needs to be proven by subsequent studies on prospectively bioptically gathered specimens in the clinical setting.

As mentioned morphologic heterogeneity in lung cancer can be high and the amount of tumor tissue in biopsies low [35]. Therefore, not only tissue sparing techniques, as presented here, are needed but these must also deliver accurate and valid diagnostic information. Intratumoral heterogeneity of conventional growth patterns and IHC stains was evaluated by calculating kappa-statistics for the three TMA cores of each tumor. Especially for those tumors which lacked distinct growth patterns in at least one core, a considerably higher concordance measured by Cohen's kappa for IHC supported classification, in comparison to conventional H&E typing was observed. In concordance with our findings, Mukhopadhyay et al. have recently described that immunohistochemical variances between biopsies and resection specimens are uncommon [7]. This further supports our finding that variations in IHC expression patterns are less frequent than in histological growth patterns. Combined morphologic-IHC classification, especially in small biopsies, should, therefore, be recommended and stipulated, especially knowing the therapeutic consequences.

In a large study, Sculier et al could detect squamous morphology in NSCLC as a prognostic factor in UICC stage IIIA patients, only [38]. Other histological entities or squamous differentiation in other cancer stages were of no prognostic significance. This is also reflected in our cohort using conventional H&E classification of NSCLC into LAC, SCC, LCC and LCNEC which was performed on resection specimens. On the other hand, using our multi-antibody assay in conjunction with the slightly modified diagnostic algorithm proposed by Travis et al. [9], distinct separation of Kaplan-Meier survival plots is evident and pointing towards a statistical trend. Our cohort consists not only of a considerable number of patients, but also covers almost two decades of NSCLC with long term follow-up. Thus, biological features of lung carcinomas are to be reflected in these survival plots according to cancer's biology. It can, therefore, most probably be assumed that histological subentities of NSCLC, especially LAC, SCC and LCNEC, not only display different morphologies, but also inhabit different biological properties. This is also reflected in our survival analysis where overall survival significantly differed between the therapeutically relevant subgroups of LAC+NSCLC NOS, SCC and LCNEC. Thus, making a subtle and accurate diagnosis of each entity is essential for current and even more future personalized therapeutic decision making. Nevertheless, additional studies using analog IHC classification algorithms including the possibility for diagnosing LCNEC and based upon prospective typing of lung biopsies will be needed to further verify this finding of ours.

Concluding, we here for the first time demonstrate that tissue sparing IHC analyses for subtyping of NSCLC using a simultaneous 6-antibody assay for assessment not only of lineage differentiation but also including LCNEC is feasible and helps to make valid and accurate tissue based diagnoses. The use of our multi-antibody staining spares extremely limited tissue samples, especially if dealing with biopsy material. Additionally, this multi-antibody assay saves substantial economic and diagnostic resources. Furthermore, we are the first to demonstrate that additional IHC investigations better reflect the biology of NSCLC subtypes especially with regard to LCNEC than conventional H&E classification. Combined morphological-IHC classification of NSCLC should, therefore, be included in routine histopathological diagnostic pathways to assure accurate treatment of NSCLC patients.

## Supporting Information

Document S1 **Detailed immunohistochemical staining protocol established for DAKO autostainer.**
(DOCX)

Figure S1 **Multi-antibody protocol without second primary antibody cocktail reveals no chromogen cross reaction.** The complete multi-antibody assay was applied with exception of application of the second primary antibody set (p63, CD56, Chromogranin A, Synaptophysin). As no specific brow coloration is evident, it can be concluded, that there are no interferences of the second HRP/DAB-based detection system with the first primary antibody set (Vimentin, TTF1). A) Only TTF1 was applied as first primary antibody. B) Only Vimentin was applied as first primary antibody.
(TIF)

Figure S2 **Multi-antibody assay with delicate appreciation of double positivity.** As well TTF1/p63 double positive cells (A), as well as Vimentin/NE-marker double positive cells (B) can be accurately assessed by our multi-antibody assay. “*” marks exemplarily areas of double positive cells.
(TIF)

Figure S3 **Forest plot of odd's ratios in favour of squamous (A) and glandular (B) differentiation.** In each single TMA cores A to C p63 and TTF1 give excellent separation of both differentiation lines reflecting their usefulness for separating LAC and SCC.
(TIF)

Figure S4 **ROC-AUC plots for diagnostic reliability of each IHC marker.** Concordance with the H&E classification of the resection specimen (top) and the combined IHC classification algorithm (bottom) was calculated for each entity. In LAC and SCC p63 and TTF1 reveal high values for sensitivity and specificity. Vimentin does not show significant diagnostic value, whereas neuroendocrine markers in combination with p63 are useful for diagnosing LCNEC.
(TIF)

Figure S5 **Kaplan-Meier survival curves with regard to expression of neuroendocrine markers.** Analyses of the whole cohort (A), as well as of the NSCLC entities LAC (C) and SCC (D) do not reveal any influence of expression of neuroendocrine (NE) markers on overall survival. On the other hand, tumors classified by the combined morphology-IHC algorithm as NSCLC NOS (B) showed a statistical trend towards a poorer outcome if NE markers could be immunohistochemically detected (p = 0.095).
(TIF)

Table S1 **Summary of AUC values for each tested marker in relation to the subtypes of NSCLC.** TTF1 and p63 prove to be of highly significant value for evaluation of linage differentiation.
(DOC)
