# Supplementary material for: Simultaneous Multi-Antibody Staining in Non-Small Cell Lung Cancer Strengthens Diagnostic Accuracy Especially in Small Tissue Samples
Source: PLoS One. 2013 Feb 13;8(2):e56333. doi: 10.1371/journal.pone.0056333 (PMC3572034; doi:10.1371/journal.pone.0056333)
Supplement: Document S1 — Detailed immunohistochemical staining protocol established for DAKO autostainer. (DOCX) [file pone.0056333.s001.docx]

**Detailed immunohistochemical staining protocol established for DAKO autostainer**

*Antigen retrieval:*

heat induced antigen retrieval with citrate buffer pH 6.1 at 120°C using a pressure cooker for 2 minutes

*Step by step protocol on DakoAutostainer:*

- Rinse with Dako Wash Buffer
- First primary antibody cocktail: mouse-anti human vimentin (1:500; Dako;Catalognumber:M7020; clone VIM3B4/1) and mouse-anti-human TTF1 (1:200;Dako, Catalognumber: M3575,clone 8G7G3). Incubation time 30 minutes
- Rinse with Dako Wash Buffer
- Secondary antibody: Dako REAL link biotinylated secondary antibody (AB2). Incubation time 15 minutes
- Rinse with Dako Wash Buffer
- Activation: Dako REAL Alkaline Phosphatase/RED Streptavidin Alkaline Phosphatase (AP). Incubation time 15 minutes
- Visualization: Dako REAL Chromogen (RED). Incubation time 10 minutes.
- Rinse with Dako Wash Buffer
- Rinse with Dako Wash Buffer
- Blocking reagent: EnVsion FLEX Peroxidase-Blocking Reagent. Incubation time 5 minutes.
- Rinse with Dako Wash Buffer
- Second primary antibody cocktail: mouse anti-human p63 (1:100; DCS Innovative Diagnostik Systems, Catalognumber: PI627C02; clone 4A4), mouse anti-human chromogranin (1:200; Dako; Catalognumber: M0869; clone DAK-A3), mouse anti-human synaptophysin (1:200; DCS Innovative Diagnostik Systems; Catalognumber: SI682C01; clone SY38) and mouse anti-human CD56 (1:100; Novocastra; Catalognumber: NCL-L-CD56-1B5; clone 1B6). Incubation time 30 minutes.
- Rinse with Dako Wash Buffer
- Secondary antibody coupled with activation reagent: EnVision FLEX HRP. Incubation time 20 minutes
- Rinse with Dako Wash Buffer
- Visualization: EnVision FLEX DAB+ Chromogen. Incubation time 10 minutes
- Rinse with Dako Wash Buffer

*Counterstain* with hematoxylin manually.

Dehydration in ascending alcohol row

Coverlip
