# Supplementary figures and images for: Simultaneous Multi-Antibody Staining in Non-Small Cell Lung Cancer Strengthens Diagnostic Accuracy Especially in Small Tissue Samples
Source: PLoS One. 2013 Feb 13;8(2):e56333. doi: 10.1371/journal.pone.0056333 (PMC3572034; doi:10.1371/journal.pone.0056333)

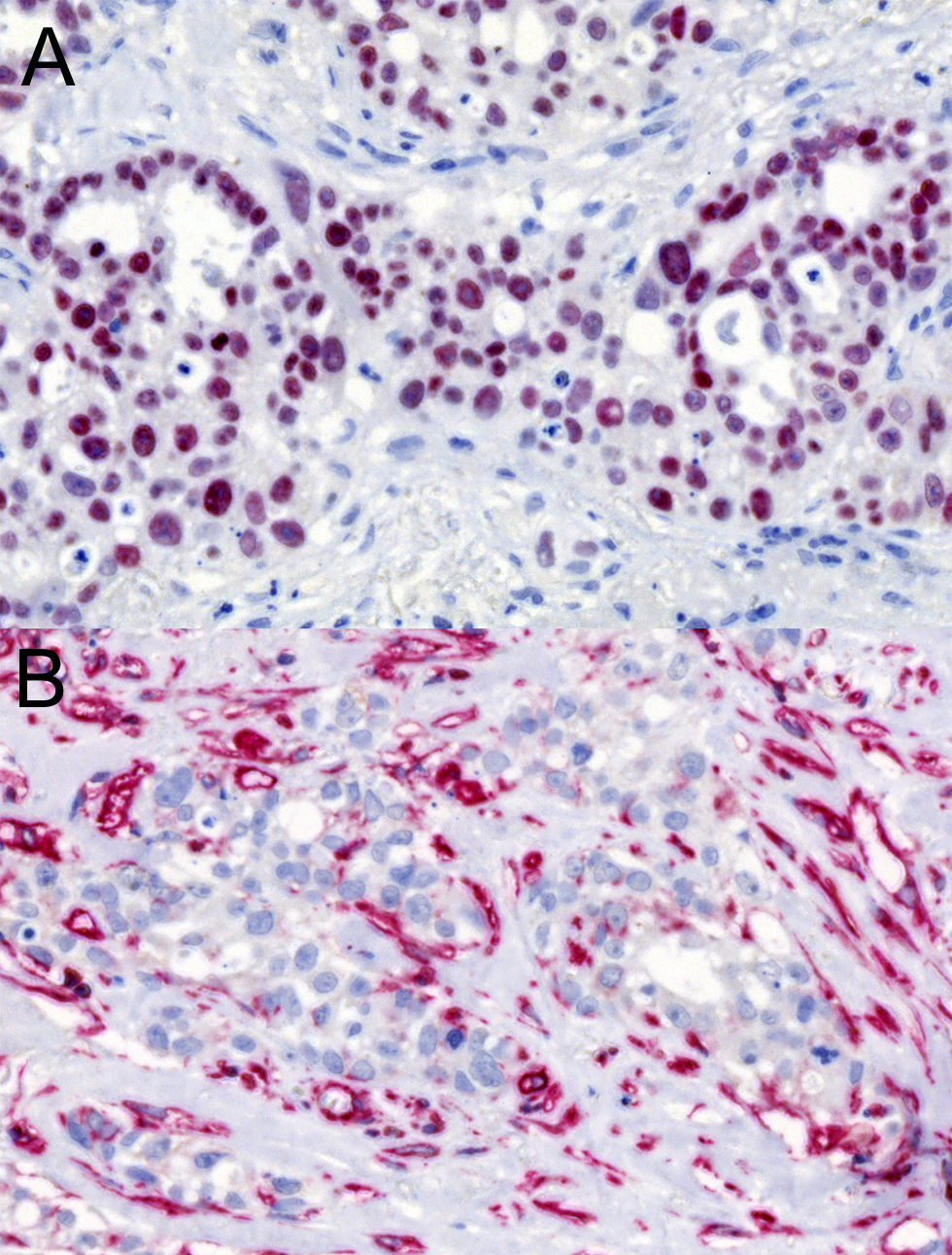

Supplement: Figure S1 — Multi-antibody protocol without second primary antibody cocktail reveals no chromogen cross reaction. The complete multi-antibody assay was applied with exception of application of the second primary antibody set (p63, CD56, Chromogranin A, Synaptophysin). As no specific brow coloration is evident, it can be concluded, that there are no interferences of the second HRP/DAB-based detection system with the first primary antibody set (Vimentin, TTF1). A) Only TTF1 was applied as first primary antibody. B) Only Vimentin was applied as first primary antibody. (TIF) [file pone.0056333.s002.tif]

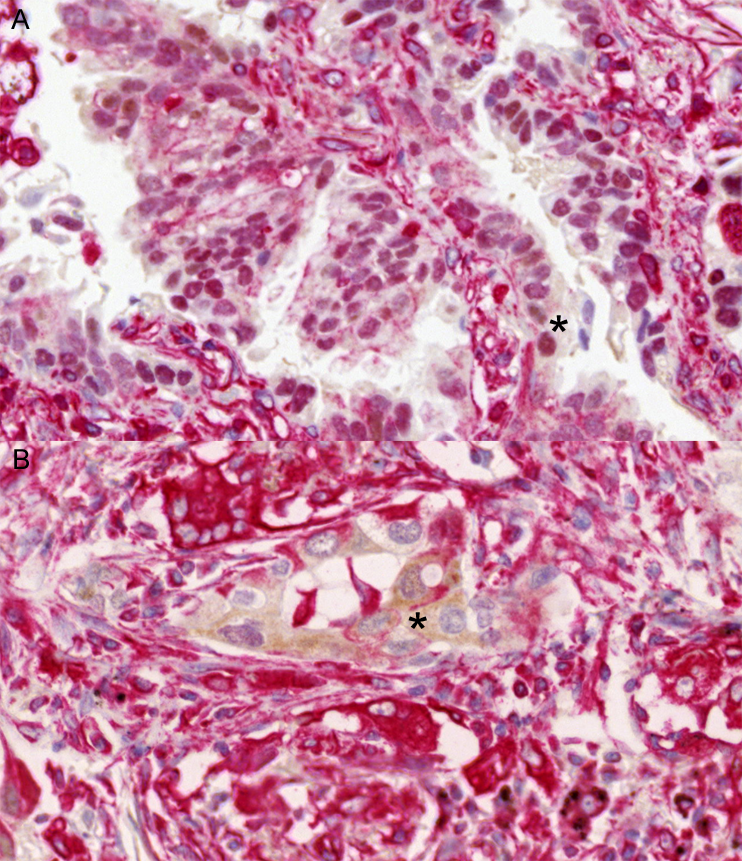

Supplement: Figure S2 — Multi-antibody assay with delicate appreciation of double positivity. As well TTF1/p63 double positive cells (A), as well as Vimentin/NE-marker double positive cells (B) can be accurately assessed by our multi-antibody assay. “*” marks exemplarily areas of double positive cells. (TIF) [file pone.0056333.s003.tif]

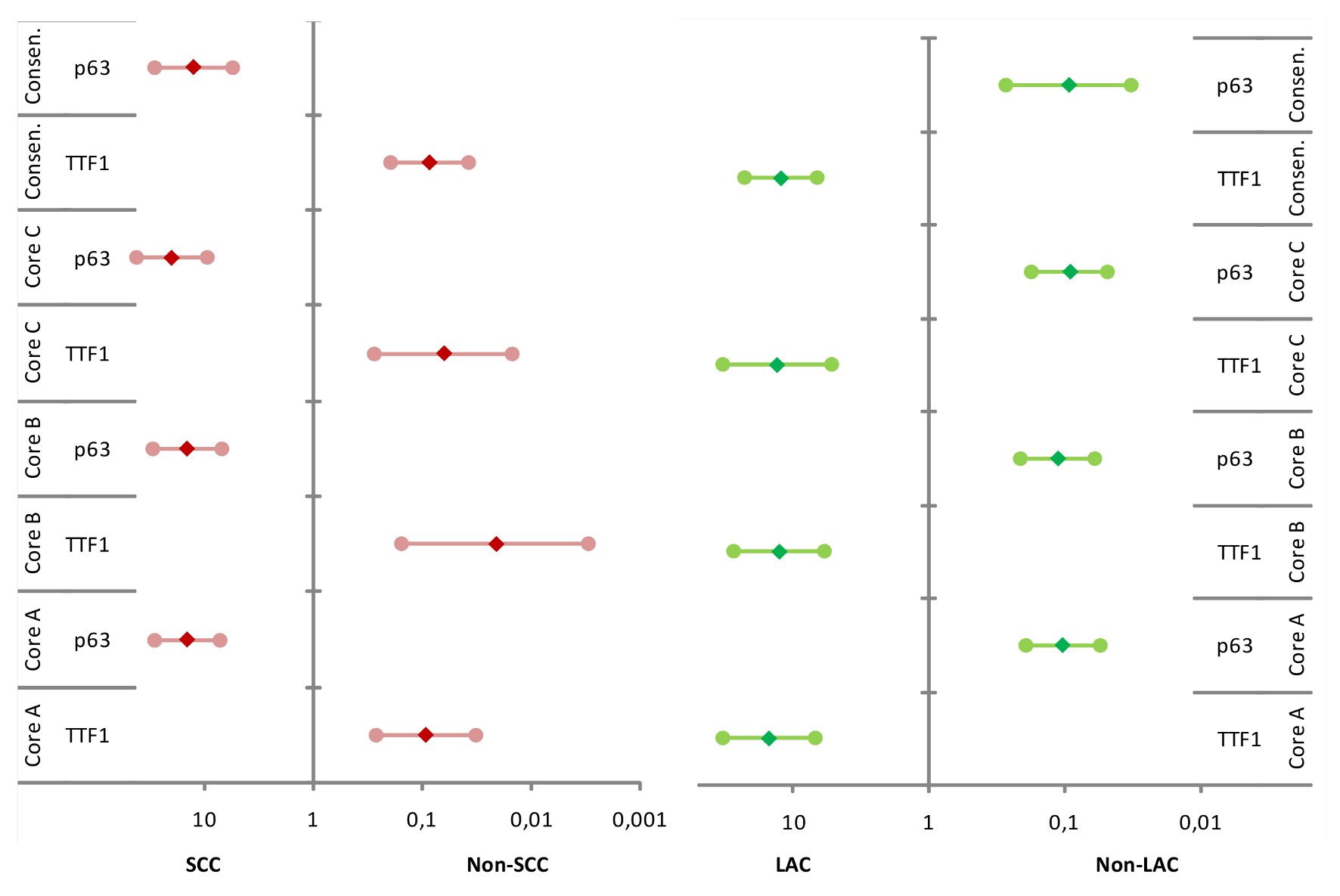

Supplement: Figure S3 — Forest plot of odd's ratios in favour of squamous (A) and glandular (B) differentiation. In each single TMA cores A to C p63 and TTF1 give excellent separation of both differentiation lines reflecting their usefulness for separating LAC and SCC. (TIF) [file pone.0056333.s004.tif]

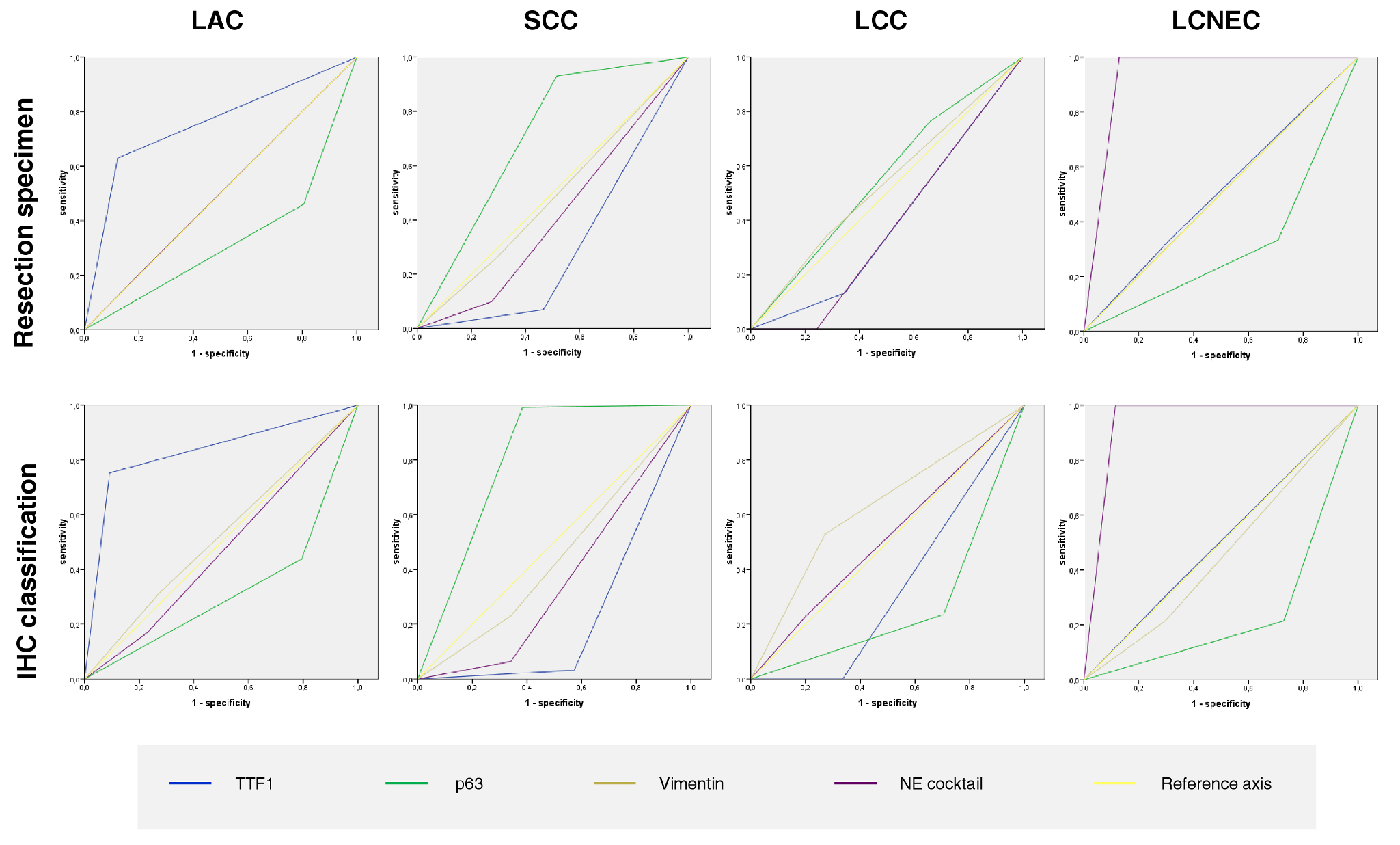

Supplement: Figure S4 — ROC-AUC plots for diagnostic reliability of each IHC marker. Concordance with the H&E classification of the resection specimen (top) and the combined IHC classification algorithm (bottom) was calculated for each entity. In LAC and SCC p63 and TTF1 reveal high values for sensitivity and specificity. Vimentin does not show significant diagnostic value, whereas neuroendocrine markers in combination with p63 are useful for diagnosing LCNEC. (TIF) [file pone.0056333.s005.tif]

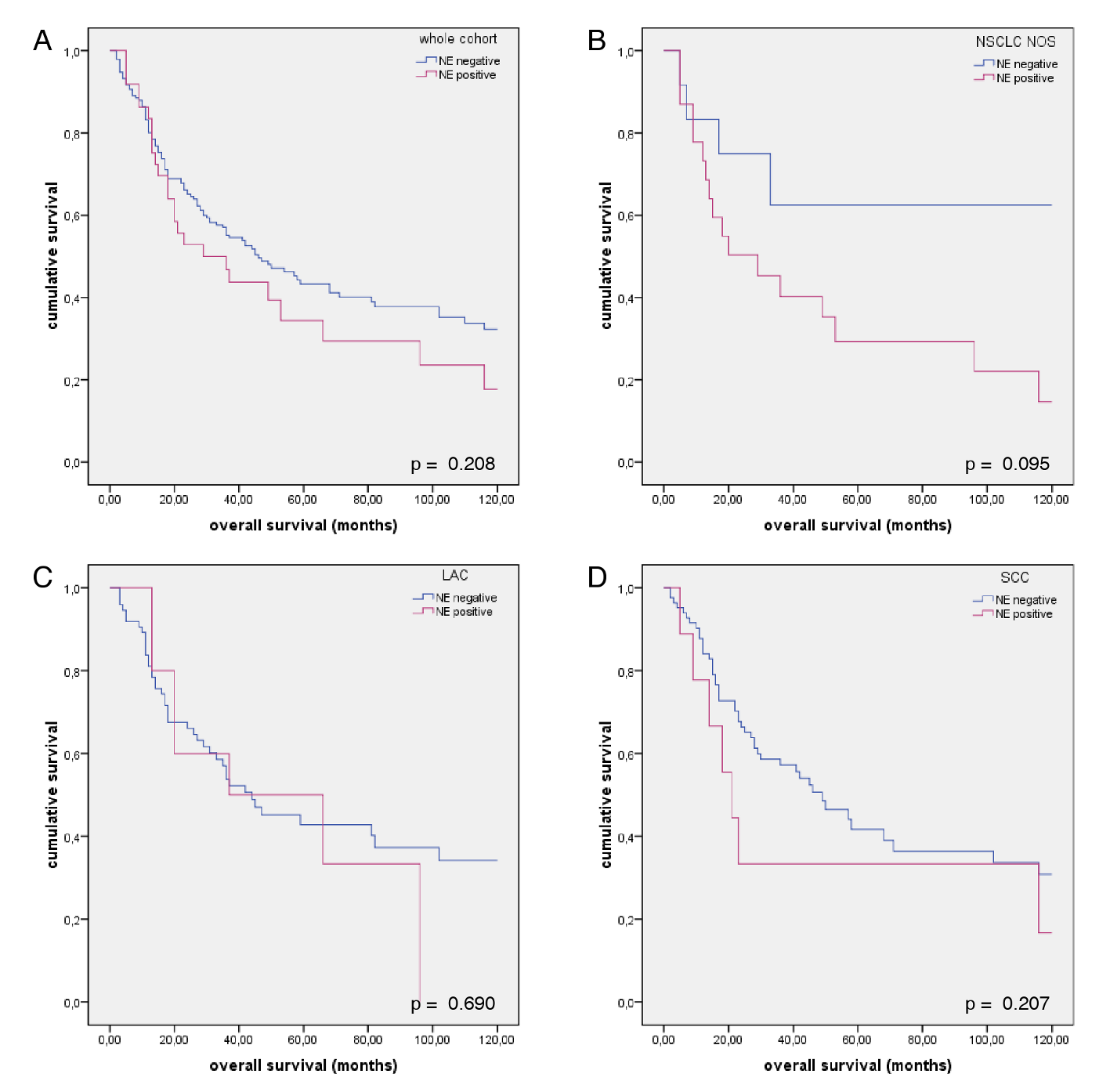

Supplement: Figure S5 — Kaplan-Meier survival curves with regard to expression of neuroendocrine markers. Analyses of the whole cohort (A), as well as of the NSCLC entities LAC (C) and SCC (D) do not reveal any influence of expression of neuroendocrine (NE) markers on overall survival. On the other hand, tumors classified by the combined morphology-IHC algorithm as NSCLC NOS (B) showed a statistical trend towards a poorer outcome if NE markers could be immunohistochemically detected (p = 0.095). (TIF) [file pone.0056333.s006.tif]
