# Supplementary material for: Simultaneous Multi-Antibody Staining in Non-Small Cell Lung Cancer Strengthens Diagnostic Accuracy Especially in Small Tissue Samples
Source: PLoS One. 2013 Feb 13;8(2):e56333. doi: 10.1371/journal.pone.0056333 (PMC3572034; doi:10.1371/journal.pone.0056333)
Supplement: Table S1 — Summary of AUC values for each tested marker in relation to the subtypes of NSCLC. TTF1 and p63 prove to be of highly significant value for evaluation of linage differentiation. (DOC) [file pone.0056333.s007.doc]

**Table S1: Summary of AUC values for each tested marker in relation to the subtypes of NSCLC**. TTF1 and p63 prove to be of highly significant value for evaluation of linage differentiation.

|  |  | **TTF1** | | | **p63** | | | **Vimentin** | | | **NE-cocktail** | | |
| --- | --- | --- | --- | --- | --- | --- | --- | --- | --- | --- | --- | --- | --- |
|  |  | AUC | 95 % KI | p-value | AUC | 95 % KI | p-value | AUC | 95 % KI | p-value | AUC | 95 % KI | p-value |
| **Resection specimen** | **LAC** | 0.754 | 0.69 – 0.82 | ***< 0.001*** | 0.328 | 0.26 – 0.40 | ***< 0.001*** | 0.502 | 0.43 – 0.57 | 0.963 | 0.485 | 0.41 – 0.56 | 0.692 |
|  | **SCC** | 0.300 | 0.24 – 0.36 | ***< 0.001*** | 0.710 | 0.65 – 0.77 | ***< 0.001*** | 0.481 | 0.41 – 0.55 | 0.606 | 0.427 | 0.36 – 0.50 | 0.045 |
|  | **LCC** | 0.393 | 0.31 – 0.48 | 0.035 | 0.552 | 0.46 – 0.65 | 0.306 | 0.532 | 0.43 – 0.63 | 0.532 | 0.396 | 0.31 – 0.48 | 0.040 |
|  | **LCNEC** | 0.510 | 0.39 – 0.63 | 0.866 | 0.313 | 0.20 – 0.43 | *0.002* | 0.502 | 0.38 – 0.62 | 0.973 | 0.906 | 0.84 – 0.97 | *< 0.001* |
|  |  |  |  |  |  |  |  |  |  |  |  |  |  |
| **IHC-classification** | **LAC** | 0.834 | 0.78 – 0.89 | **< 0.001** | 0.320 | 0.25 – 0.39 | **< 0.001** | 0.526 | 0.45 – 0.60 | 0.488 | 0.447 | 0.38 – 0.52 | 0.158 |
|  | **SCC** | 0.230 | 0.17 – 0.29 | **< 0.001** | 0.816 | 0.76 – 0.87 | **< 0.001** | 0.460 | 0.39 – 0.53 | 0.268 | 0.377 | 0.31 – 0.45 | *0.001* |
|  | **LCNEC** | 0.496 | 0.39 – 0.61 | 0.938 | 0.235 | 0.15 – 0.32 | *< 0.001* | 0.457 | 0.35 – 0.56 | 0.439 | 0.963 | 0.94 – 0.98 | *< 0.001* |
|  | **NSCLC** | 0.338 | 0.22 – 0.45 | 0.049 | 0.269 | 0.13 – 0.41 | *0.005* | 0.678 | 0.52 – 0.84 | 0.030 | 0.405 | 0.27 – 0.54 | 0.248 |
